# Community Emergency Department Management of a Nail Gun Puncture Wound: A Case Report

**DOI:** 10.7759/cureus.87831

**Published:** 2025-07-13

**Authors:** Daniel McDermand, Jacqueline M Dovgalyuk

**Affiliations:** 1 Emergency Medicine, UTHSC (University of Tennessee Health Science Center) Nashville - St. Thomas Health, Murfreesboro, USA

**Keywords:** community emergency department, nail gun injury, occupational hand injury, penetrating hand injury, plastic hand surgery

## Abstract

Nail gun injuries, generally accidental, result in puncture wounds usually involving the hands and fingers, and such injuries are common in both urban and rural settings. Recognition of the extent of soft tissue injury, establishing hemostasis, assessing for bony, tendinous, or neurovascular injury, and consideration of infection prophylaxis and pain control are key components of emergency department (ED) workup. This report describes the management of a 51-year-old male who presented to a community setting ED with a chief complaint of left hand pain after sustaining a nail gun injury just prior to arrival. We will address immediate management, ED treatment, and ultimate disposition, as well as management strategies for patients who present with similar complaints to a community-setting ED.

## Introduction

Nail gun injuries are common, especially in the acute care setting. Many nail gun injuries can be appropriately managed in the emergency department. This case provides insight regarding the management of a nail gun injury to the hand in a community-setting emergency department (ED), without direct access to or availability of specialists, including hand or plastic surgery. Specifically, this report addresses the management of a 51-year-old male with a nail gun injury to his left hand.

## Case presentation

A 51-year-old, right-hand-dominant male with a history of diabetes and prior hospital admission due to septic bursitis presented to the ED with a complaint of left hand pain. The patient stated that he was using a nail gun just prior to arrival when he accidentally shot a nail through his left hand. He complained of pain around the puncture sites but denied any numbness or tingling in his hand or fingers. He was still able to range all of his digits but did have some pain with range of motion (ROM). His tetanus status was up to date, with his last booster administered two to three years prior.

**Figure 1:**
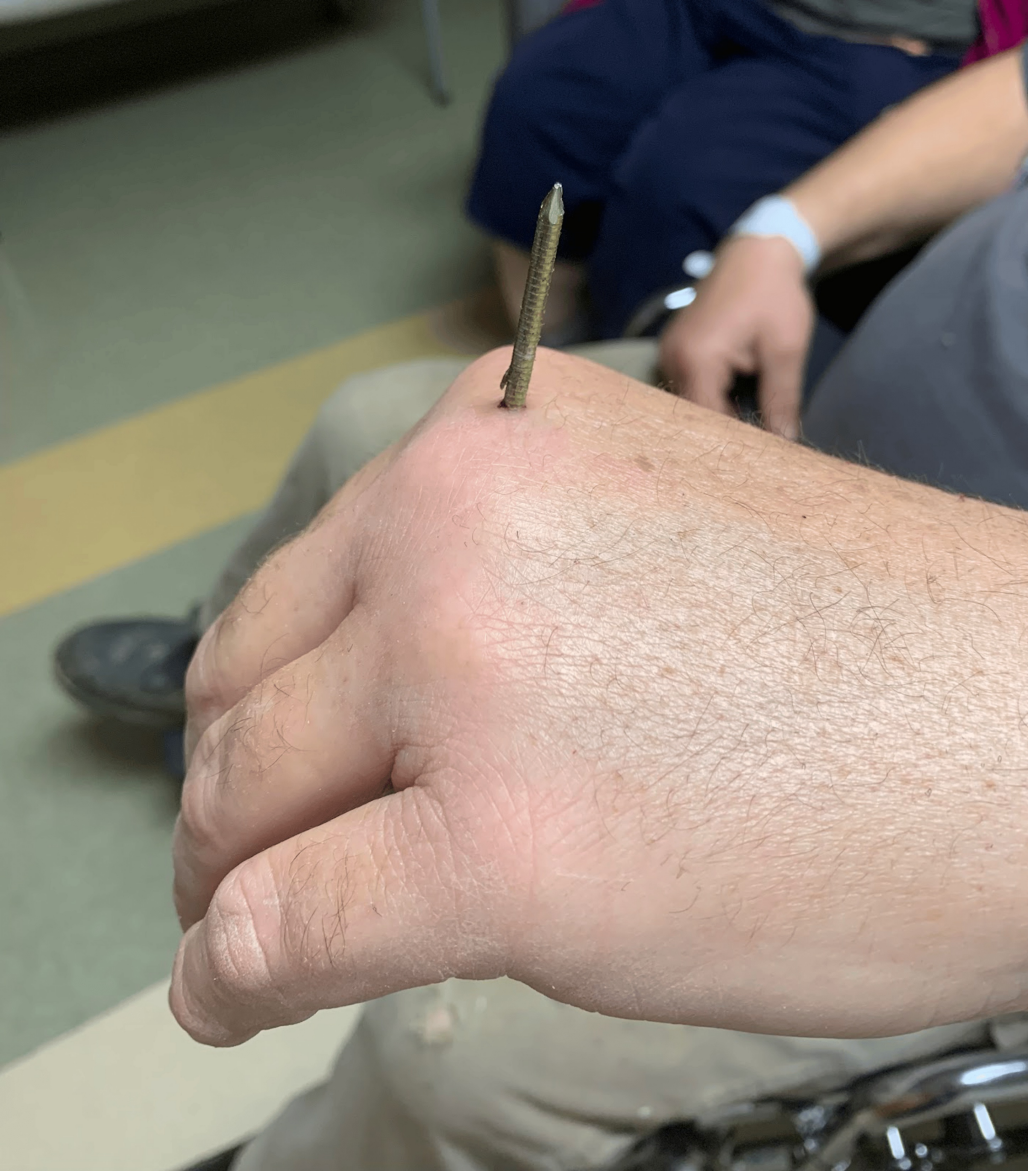
Dorsal aspect with nail position Image highlighting the position of the nail as well as the orientation of the barbs on the nail shaft.

**Figure 2:**
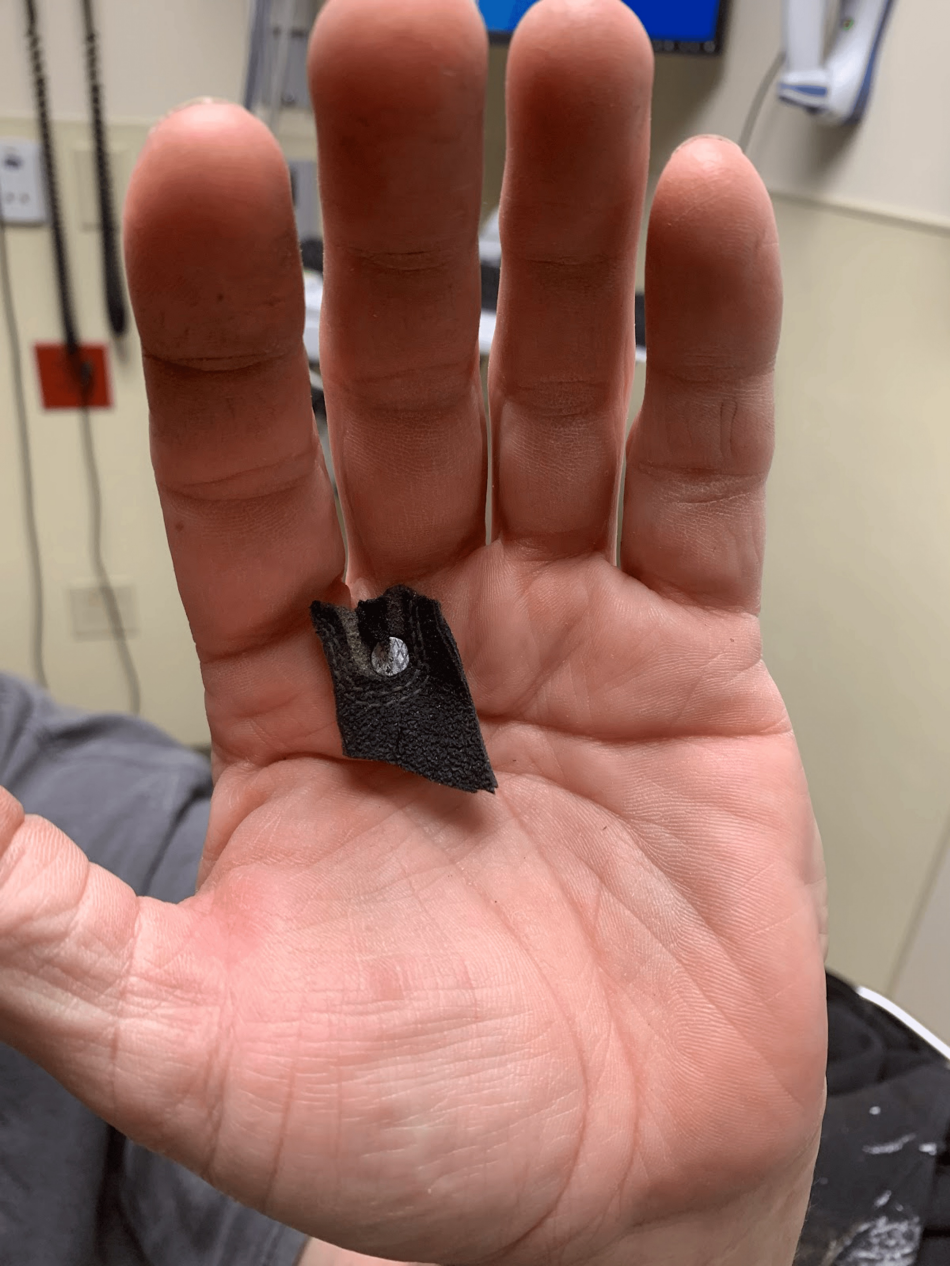
Palmar aspect with nail position Image highlighting the palmar aspect of the injury, the location between the 2nd and 3rd metacarpals, as well as the nail head, which needed to be cut off to accommodate removal from the dorsal aspect.

His initial vitals were stable: temperature of 97.5 °F (oral), heart rate 75 beats per minute, blood pressure of 167/107 mmHg, and oxygen saturation of 94% on ambient air. His exam was notable for a 3-inch nail extending from the palmar surface through the dorsal aspect of the left hand, in between the second and third metacarpals (Figures 1, 2), normal ROM of digits, neurovascularly intact with a normal radial pulse, and no active bleeding from the puncture wound sites.

**Figure 3:**
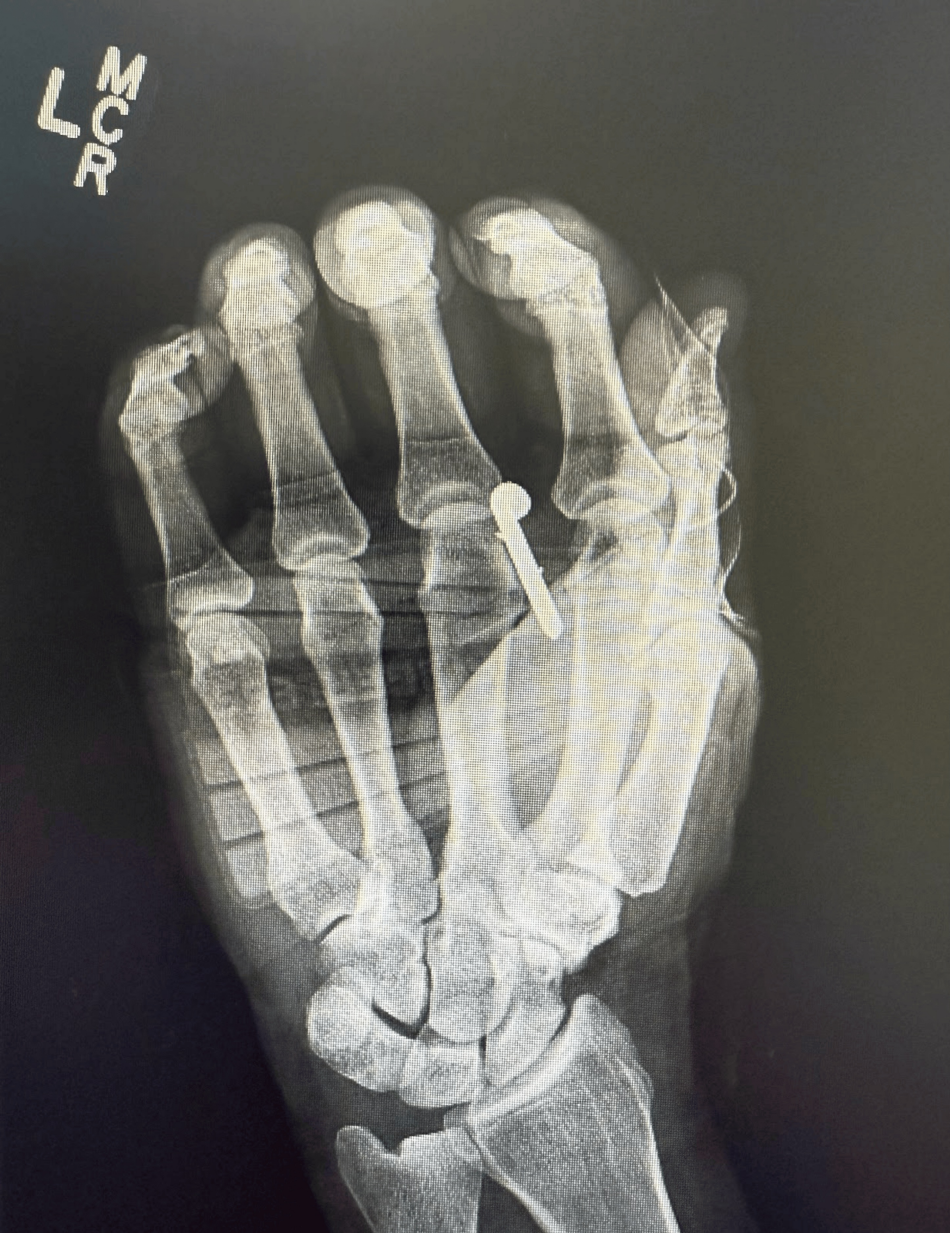
Anterior-posterior X-ray with nail position X-ray image showing the nail traversing between the 2nd and 3rd digits without evidence of associated bony injury.

**Figure 4:**
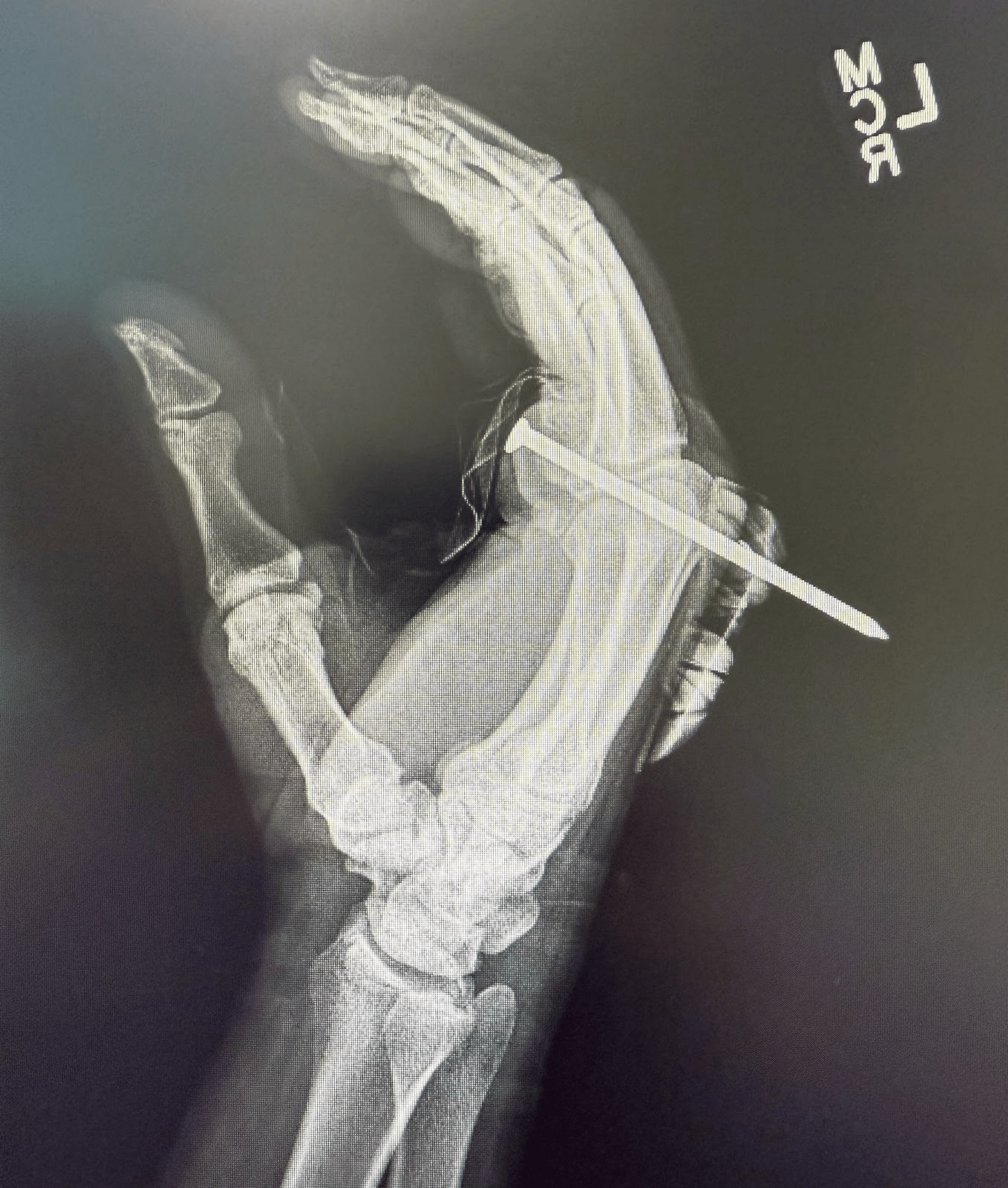
Lateral X-ray image X-ray image highlighting the orientation of the nail.

Given the nature and location of the injury, plain films of the area were obtained to evaluate for bony involvement. X-rays of the left hand showed the nail traversing the hand through the level of the distal metacarpals, extending between the second and third metacarpals, without evidence of fracture or joint dislocation (Figures 3, 4). The patient was given oral medication for pain control.

**Figure 5:**
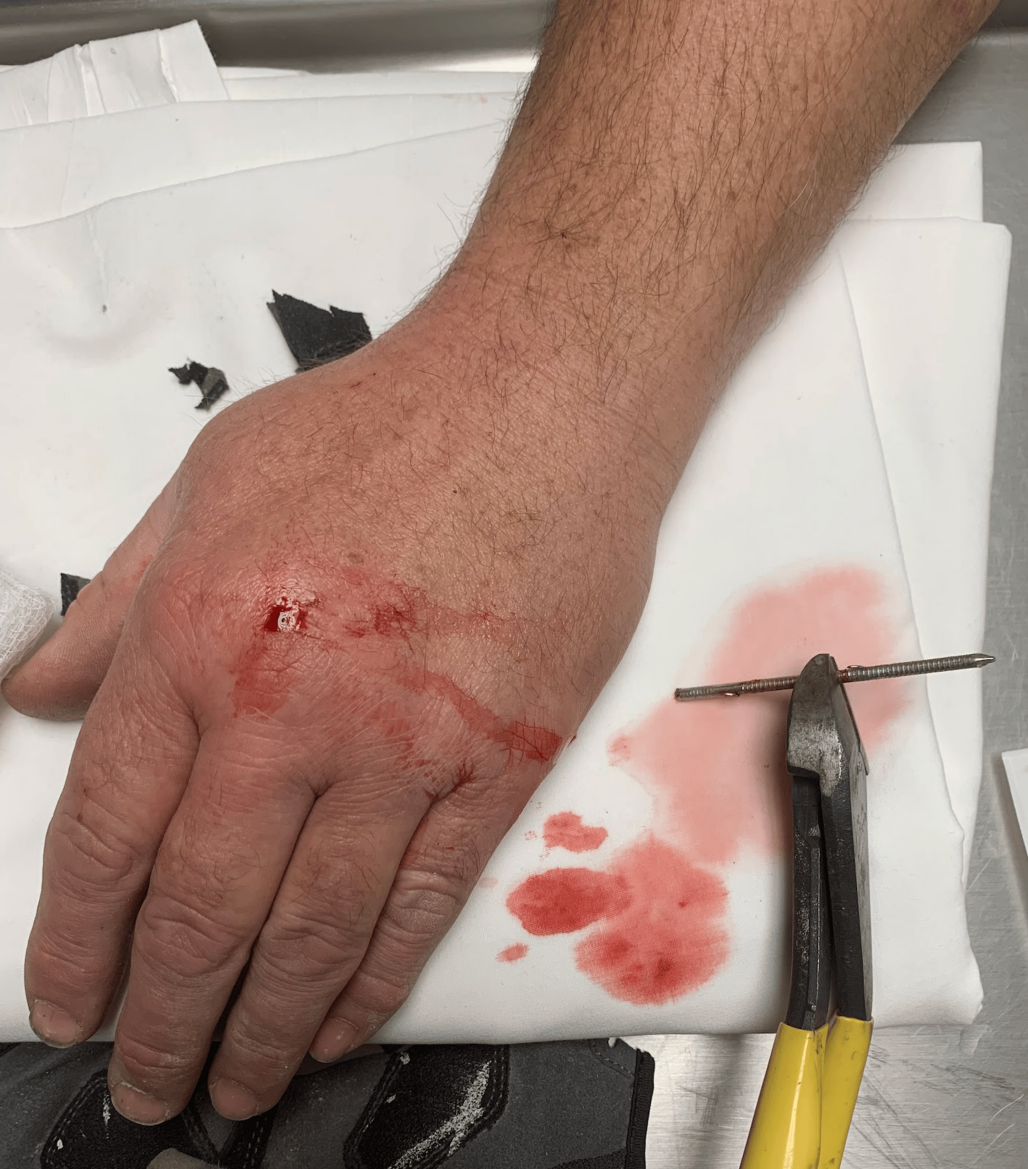
Nail after removal with wire cutters Image showing the head of the nail cut off prior to removal from the dorsal aspect of the hand.

Prior to any attempts at removal, plastic surgery was consulted from the ED. Plain films did not show significant bony or soft tissue damage, and given reassuring exam findings, the plastic surgeon on call recommended and agreed with removal of the nail in the emergency department, with instructions to follow up in the clinic setting. The nail was barbed, with the barbs oriented toward the palmar aspect of the patient’s hand. Thus, the top of the nail was cut off with wire cutters and the area was thoroughly anesthetized with local lidocaine. Utilizing the dorsum of the patient’s hand as a fulcrum, the nail was removed without difficulty from the dorsal aspect (Figure 5). There was minimal bleeding noted after removal, easily controlled with direct pressure to the dorsal puncture wound site. Following removal, the puncture wound sites were copiously irrigated with sterile saline.

The patient was ultimately discharged from the ED with prescriptions for Keflex 500 mg BID for 10 days and tramadol 50 mg PRN as needed for pain (eight tablets). The decision to prophylactically treat with antibiotics was made given the patient’s underlying history of diabetes and prior hospital admission due to septic bursitis. He was given instructions to follow up with plastic surgery in the outpatient setting and return to the ED for any new or worsening symptoms such as fever, redness, warmth, drainage, or significant pain. The patient did not return to the ED.

## Discussion

Nail gun injuries, generally accidental, result in puncture wounds usually involving the hands and fingers. From 2001 to 2005, it was estimated that nail gun injuries accounted for 37,000 ED visits each year [1]. Management of these injuries can generally be accomplished in the ED. This case highlights management in a community ED setting. Despite the intricate anatomy of the hand, one series reported that only 25% of isolated nail gun injuries to the hand resulted in significant structural damage, such as tendon injury, joint space violation, or nerve injury [2].

From a community ED perspective, careful examination and consideration of possible surgical exploration must be undertaken prior to removal attempts. In this particular case, the plain films confirmed no underlying bony involvement. The patient did not exhibit brisk bleeding suggestive of arterial injury, nor did he have any significant neurovascular compromise on exam. As such, despite not having specialist support on site, the plastic surgeon available on call recommended and agreed with removal of the nail in the ED.

The nail was barbed, with the orientation of the barbs allowing for removal only from the dorsal aspect so as to minimize the risk of iatrogenic injury during extraction. After removal of the top of the nail with wire cutters, both the palmar and dorsal puncture sites were injected liberally with about 10 cc of lidocaine 1% without epinephrine. The underlying deeper soft tissue surrounding the nail tract was also anesthetized.

Following local anesthetic, pliers were utilized to firmly grasp the nail from the dorsal aspect, and this case highlights a unique technique in which the patient’s hand was used as a fulcrum to facilitate smooth removal. This technique allowed the ED physician to maintain continuous contact with the patient’s hand, minimizing the risk of iatrogenic injury as the nail was slowly retracted using a lever-type motion. After removal, the puncture sites were thoroughly cleaned, bandaged, and the patient was discharged with antibiotics, pain medication, and instructions to follow up with plastic surgery in the outpatient setting.

In similar community ED settings without hand or plastic surgery readily available, this case provides an appropriate strategy for ED workup, non-surgical intervention, and disposition. Nail gun injuries have the potential to cause significant structural and neurovascular compromise, making a stepwise approach to these types of traumatic injury critical. Confirm lack of bony injury with x-ray imaging prior to procedural intervention, visualize orientation of the nail as well as any barbs (which may have implications regarding direction of removal), use lidocaine to anesthetize the puncture wounds and wound tract, and if possible, utilize the patient’s hand (or other puncture site) as a fulcrum when removing the nail with pliers.

In general, nail gun injuries that lack articular or neurovascular involvement can be managed with simple extraction, local wound care, and, in most cases, outpatient oral antibiotics [2]. A thorough physical exam is of utmost importance, as nonoperative methods are generally appropriate in the removal of nails not associated with neurovascular compromise, fractures, or tendon injury [3].

## Conclusions

Despite a somewhat daunting initial presentation, this case shows that nail gun injuries, when approached step by step, can be managed appropriately in a community ED setting without specialist support directly available. Key components prior to removal attempts include a thorough physical exam and X-ray imaging. Generally, nonoperative methods are appropriate for removal of nails that are not associated with neurovascular compromise, fractures, or tendon injury. If the nail gun injury has associated bony damage, tendon injury, brisk arterial bleeding, pallor, loss of pulses, or neurologic deficits on exam, nail removal should not be attempted in the ED. Imaging should always be utilized to confirm nail orientation, other characteristics of the nail (such as barbs), and to assess for underlying bony injury. If barbs are visualized, the nail must be removed in the appropriate orientation to minimize the risk of iatrogenic injury. If available, review the images and management expectations with plastic or hand surgery. If removal in the ED is feasible, ensure adequate local anesthesia, and if possible, utilize the technique demonstrated in this case of using the patient’s hand (or other puncture site) as a fulcrum to facilitate smooth removal. Following the procedure, thoroughly irrigate, ensure hemostasis, provide local wound care as needed, and determine whether the patient would benefit from prophylactic antibiotics in the outpatient setting.

## References

[1] Nail-gun injuries treated in emergency departments - United States, 2001-2005. https://www.cdc.gov/mmwr/preview/mmwrhtml/mm5614a2.htm.

[2] Pierpont YN, Pappas-Politis E, Naidu DK, Salas RE, Johnson EL, Payne WG (2008). Nail-gun injuries to the hand. Eplasty.

[3] Kordell J, Cogburn J, Khodaee M (2023). Non-operative management of an accidental knee intra-articular nail. Cureus.

